# Contrast variation by dynamic nuclear polarization and time-of-flight small-angle neutron scattering. I. Application to industrial multi-component nanocomposites^1^

**DOI:** 10.1107/S1600576716016472

**Published:** 2016-11-08

**Authors:** Yohei Noda, Satoshi Koizumi, Tomomi Masui, Ryo Mashita, Hiroyuki Kishimoto, Daisuke Yamaguchi, Takayuki Kumada, Shin-ichi Takata, Kazuki Ohishi, Jun-ichi Suzuki

**Affiliations:** a Institute of Quantum Beam Science, Ibaraki University, Ibaraki, 316-8511, Japan; b Sumitomo Rubber Industries Ltd, Kobe, 651-0072, Japan; c Materials Science Research Center, Japan Atomic Energy Agency, Ibaraki, 319-1195, Japan; d J-PARC Center, Japan Atomic Energy Agency, Ibaraki, 319-1195, Japan; e Neutron Science and Technology Center, Comprehensive Research Organization for Science and Society (CROSS), Ibaraki, 319-1106, Japan

**Keywords:** small-angle neutron scattering (SANS), contrast variation, dynamic nuclear polarization, ternary mixtures, nanocomposites

## Abstract

Dynamic nuclear polarization (DNP) at low temperature (1.2 K) and high magnetic field (3.3 T) was applied to a contrast variation study in small-angle neutron scattering (SANS) focusing on industrial rubber materials. By varying the scattering contrast by DNP, time-of-flight SANS profiles were obtained at the pulsed neutron source of the Japan Proton Accelerator Research Complex (J-PARC). The concentration of a small organic molecule, (2,2,6,6-tetramethylpiperidine-1-yl)oxy (TEMPO), was carefully controlled by a doping method using vapour sorption into the rubber specimens. With the assistance of microwave irradiation (94 GHz), almost full polarization of the paramagnetic electronic spin of TEMPO was transferred to the spin state of hydrogen (protons) in the rubber materials to obtain a high proton spin polarization (*P*_H_). The following samples were prepared: (i) a binary mixture of styrene–butadiene random copolymer (SBR) with silica particles (SBR/SP); and (ii) a ternary mixture of SBR with silica and carbon black particles (SBR/SP/CP). For the binary mixture (SBR/SP), the intensity of SANS significantly increased or decreased while keeping its *q* dependence for *P*_H_ = −35% or *P*_H_ = 40%, respectively. The *q* behaviour of SANS for the SBR/SP mixture can be reproduced using the form factor of a spherical particle. The intensity at low *q* (∼0.01 Å^−1^) varied as a quadratic function of *P*_H_ and indicated a minimum value at *P*_H_ = 30%, which can be explained by the scattering contrast between SP and SBR. The scattering intensity at high *q* (∼0.3 Å^−1^) decreased with increasing *P*_H_, which is attributed to the incoherent scattering from hydrogen. For the ternary mixture (SBR/SP/CP), the *q* behaviour of SANS was varied by changing *P*_H_. At *P*_H_ = −35%, the scattering maxima originating from the form factor of SP prevailed, whereas at *P*_H_ = 29% and *P*_H_ = 38%, the scattering maxima disappeared. After decomposition of the total SANS according to inverse matrix calculations, the partial scattering functions were obtained. The partial scattering function obtained for SP was well reproduced by a spherical form factor and matched the SANS profile for the SBR/SP mixture. The partial scattering function for CP exhibited surface fractal behaviour according to *q* ^−3.6^, which is consistent with the results for the SBR/CP mixture.

## Abstract 2

Contrast variation small-angle neutron scattering by dynamic nuclear polarization is applied to industrial multi-component nanocomposites.

## 1. Introduction   

Contrast variation in small-angle neutron scattering (SANS) is a very useful technique for investigating multi-component systems. For example, in a three-component (ternary) system, contrast variation can emphasize the scattering from a specific component. Therefore, by decomposing the result, we can obtain the partial scattering functions. For this purpose, deuterium substitution has conventionally been used, which takes advantage of the difference in neutron scattering length between protons and deuterons. The deuterium substitution technique can easily be applied to solutions or gels, owing to the reasonable availability of deuterated solvents, whereas the synthesis of deuterated polymers is more costly and requires greater effort, especially for industrial materials. Hence, alternative methods applicable to industrial polymer systems are needed.

**Figure 1:**
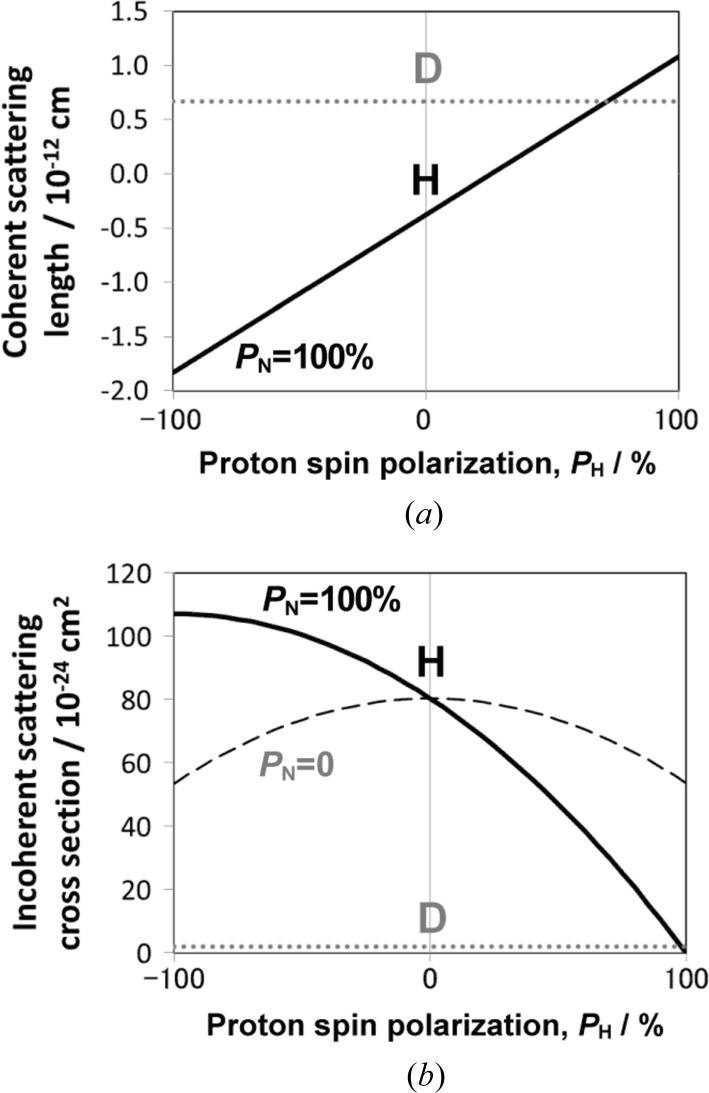
(*a*) Neutron coherent scattering length and (*b*) neutron incoherent scattering cross section of a proton as a function of proton spin polarization *P*_H_.

Besides deuterium substitution, contrast variation can also be achieved by controlling the spin states of both neutrons and protons. The coherent scattering length (*b*_coh,H_) and in­coherent scattering cross section (*σ*_inc,H_) for hydrogen (or protons) are given by the following equations (Sears, 1992 ▸):
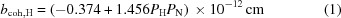
and
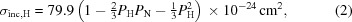
where *P*_N_ and *P*_H_ denote the polarization of neutrons and protons, respectively. Polarization is the difference in populations between up and down spins. Figs. 1 ▸(*a*) and 1 ▸(*b*) show the *P*_H_ dependence of *b*_coh,H_ and *σ*_inc,H_, respectively. Note that the variation in *b*_coh,H_ is about 2.5 times larger than that caused only by deuterium substitution under ordinary conditions (then *P*_H_ *P*_N_ = 0). For *P*_H_ *P*_N_ = 0, the coherent scattering length of hydrogen is −0.374, whereas that for a deuteron is 0.667 × 10^−12^ cm (Sears, 1992 ▸).

Polarization at thermal equilibrium (TE) states for proton (*P*_H,TE_) and electron spins (*P*_e,TE_) can be described by considering the Zeeman splitting energy and Boltzmann statistics:
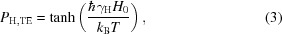


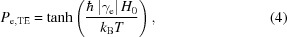
where 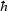 is Planck’s constant divided by 2π, γ_H_ and γ_e_ are the gyromagnetic ratios of a proton and an electron, respectively, *H*_0_ is the magnetic field, *k*_B_ is Boltzmann’s constant, and *T* is the temperature.

Table 1 ▸ lists the polarization values at thermal equilibrium evaluated according to equations (3) and (4). At room temperature, up and down proton spins are almost equally populated. With decreasing temperature, spin polarization increases. However, even at 3.3 T and 1.2 K, the proton spin is polarized up to only 0.3%. In contrast, the electron spin is polarized up to 95% under the same conditions (at 3.3 T and 1.2 K). This is because of the large difference in gyromagnetic ratio between the electron spin and the proton spin (|γ_e_|/γ_H_ = 658). The large polarization of the electron spin can be transferred to the proton spin by microwave irradiation with energy equal to the simultaneous flipping of electron and proton spins (Abragam & Goldman, 1978 ▸). Consequently, high proton spin polarization is achieved. This is called dynamic nuclear polarization (DNP) and requires electron spin doping, a magnetic field, low temperature and microwave irradiation.

For SANS investigations, proton spin polarization was first applied to structural analysis on the ribosomal protein structure in solution (Stuhrmann *et al.*, 1986 ▸). After that pioneering work, several papers reported the utilization of proton spin polarization in neutron scattering experiments (Kohgi *et al.*, 1987 ▸; Knop *et al.*, 1992 ▸; Fermon *et al.*, 1992 ▸; Grinten *et al.*, 1995 ▸; Brandt *et al.*, 2006 ▸, 2007 ▸; Noda *et al.*, 2009 ▸, 2011 ▸, 2013 ▸; Kumada *et al.*, 2010 ▸; Stuhrmann, 2015 ▸).

**Figure 2:**
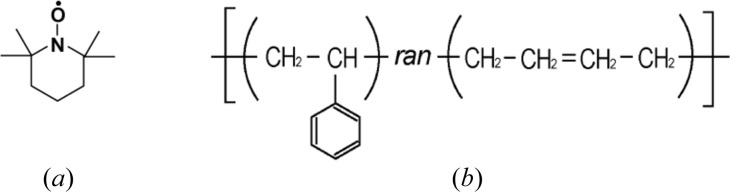
Chemical structure formulae of (*a*) TEMPO and (*b*) styrene–butadiene random copolymer, SBR.

Bunyatova (2004 ▸) originally developed the vapour sorption technique of TEMPO [(2,2,6,6-tetra­methyl­piperidine-1-yl)oxy] radicals into solid polymer materials to create polarized targets in nuclear physics experiments. Fig. 2 ▸(*a*) shows the molecular structure formula of TEMPO. On the basis of this technique, we prepared polymer systems for SANS studies after the construction of a DNP cryostat (Kumada *et al.*, 2009*a* ▸,*b* ▸) and polarized neutron ultra-small-angle scattering spectrometer (SANS-J-II) (Koizumi *et al.*, 2007 ▸) at research reactor JRR-3, Tokai, Japan. We investigated a polyethylene film (Noda *et al.*, 2009 ▸) and a di-block copolymer to evaluate precisely the inhomogeneity of the proton polarization around the doped TEMPO molecules (Noda *et al.*, 2011 ▸). Subsequently, the vapour sorption technique was successfully applied to silica-filled rubber, which is used for fuel-efficient tyres (Noda *et al.*, 2013 ▸). The vapour sorption technique can be applied to industrial rubber products after a manufacturing process.

A tyre, *i.e.* a multi-component nanocomposite, is an attractive target for contrast variation SANS with DNP. To improve wear and tear resistance, filler particles, such as carbon black (CB) and silica particles (SP), are mixed into the rubber matrix. The spatial distribution of filler particles in the rubber matrix determines not only the tyre’s reinforcement but also its energy loss performance (Schaefer *et al.*, 2000 ▸; Koga *et al.*, 2005 ▸, 2008 ▸; Takenaka *et al.*, 2009 ▸; Bouty *et al.*, 2014 ▸; Genix & Oberdisse, 2015 ▸). In accordance with the empirical knowledge that a homogeneous dispersion of filler particles lowers the energy loss, various attempts towards dispersion control have been conducted (Byers, 2002 ▸). To optimize tyre rubber performance, a reliable methodology for evaluating the filler particle dispersion is critical.

The combination of CB and SP is frequently used for manufacturing tyres. In addition to the above-mentioned effects, CB is advantageous for specific UV resistance and electric discharge. For precise structural analyses, we need to decompose the total SANS observed for a multi-component system into individual partial scattering functions.

In this article, we report our recent achievements on DNP and contrast variation SANS on model mixtures for industrial tyres. At the Materials and Life Science Experimental Facility (MLF) of the Japan Proton Accelerator Research Complex (J-PARC), we performed time-of-flight (TOF) SANS experiments, employing a wide range of neutron wavelength (λ). This causes imperfect neutron polarization (*P*_N_), depending on λ and variations in the coherent and incoherent scattering lengths.

## 2. Experimental   

### 2.1. Sample preparation   

As a model system for an industrial tyre, we prepared two types of rubber specimen: a binary mixture of styrene–butadiene random copolymer (SBR) with silica particles (SBR/SP), and a ternary mixture of SBR with silica and CB particles (SBR/SP/CP). Fig. 2 ▸(*b*) shows the molecular structure formula of SBR. As listed in Table 2 ▸, the samples consist of solution-SBR (S-SBR, Buna VSL 4720, Lanxess Corp.), silica particles (Seahostar KE-P10, Nippon Shokubai Co. Ltd), CB (N330, Tokai Carbon Co. Ltd), silane coupling agent (Si 69, Evonik Degussa GmbH) and other additives. We selected silica particles with a narrow radius distribution.

All ingredients were mixed in a milling machine and the resulting mixture was pressed into a mould and kept at 443 K for 20 min. The thicknesses of the SBR/SP and SBR/SP/CP specimens produced were 0.56 and 0.24 mm, respectively.

### 2.2. Vapour absorption of TEMPO into the rubber   

In order to perform DNP, an electron spin source is required in a specimen. By a vapour sorption technique, the stable free radical molecule TEMPO (Fig. 2 ▸ *a*) was introduced into the rubbery mixture prepared in §2.1. The vaporized TEMPO radicals were spontaneously absorbed and diffused into the amorphous matrix of SBR. We placed the rubber mixtures with TEMPO inside a sealed container at 313 K for 1 week. Consequently, the vaporized TEMPO spontaneously permeated the rubber matrix. By electron spin resonance measurements, the TEMPO concentrations were determined at 37 and 35 m*M* for the binary (SBR/SP) and ternary (SBR/SP/CP) mixtures, respectively. These concentrations were close enough to the optimum value (30 m*M*) for DNP.

The influence of the added TEMPO on the microstructure should be noted. We confirmed experimentally that SANS obtained for the mixture after TEMPO doping coincided with that obtained for the mixture before doping. The static structure, which is a target of this contrast variation SANS study, was not affected by the addition of TEMPO. On the other hand, regarding dynamic properties, the storage and loss modulus (Busfield *et al.*, 2000 ▸) and the longitudinal and transverse proton relaxation times (Stapf & Kariyo, 2005 ▸) were affected by the addition of small organic molecules (a few per cent in weight), which is known as the ‘plasticizer’ effect.

### 2.3. DNP   

**Figure 3:**
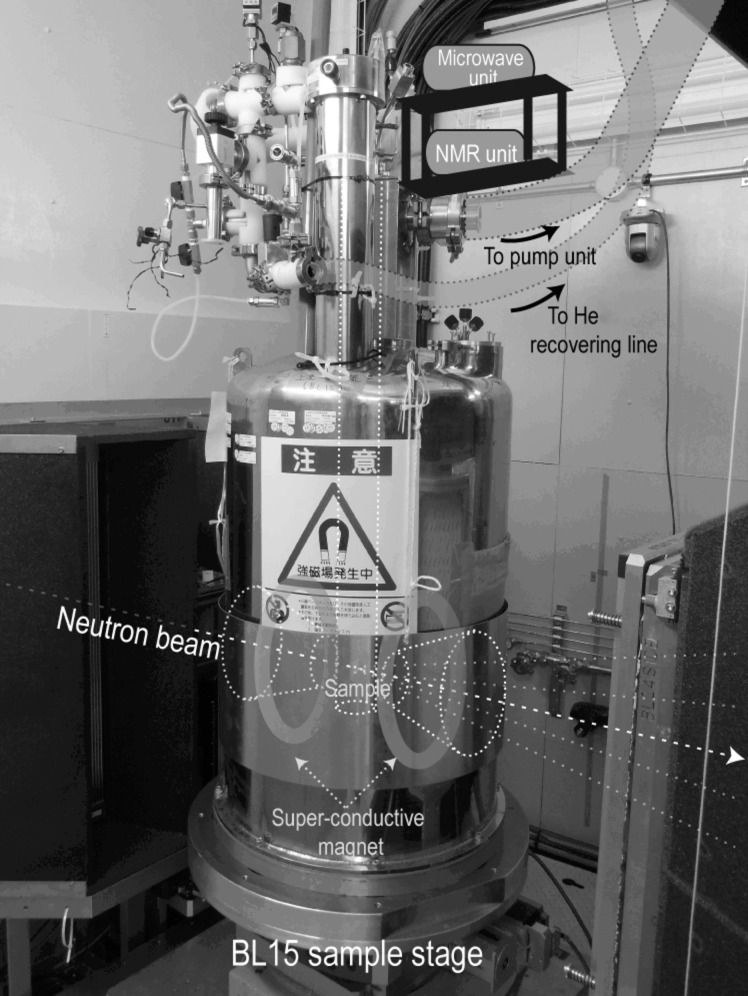
A photograph of the DNP cryostat.

Fig. 3 ▸ shows the DNP cryostat used in this study (Kumada *et al.*, 2009*a* ▸,*b* ▸). A rubber specimen is placed in a chamber filled with liquid ^4^He. By evaporating the liquid ^4^He, the specimen is cooled to 1.2 K during continuous microwave irradiation (94 GHz). The sample chamber is located between the split-type superconducting magnet coils. The superconducting magnet generates a magnetic field up to 3.5 T at the sample position. The magnetic field is parallel to the neutron beam direction. The inhomogeneity of the magnetic field (Δ*B*/*B*_0_) was designed to be less than 10^−4^ to avoid proton spin depolarization. The neutron beam passes along the central axis of the magnet coils. The windows through which the neutron beam passes are formed of thin aluminium plates, which cause less background scattering.

**Figure 4:**
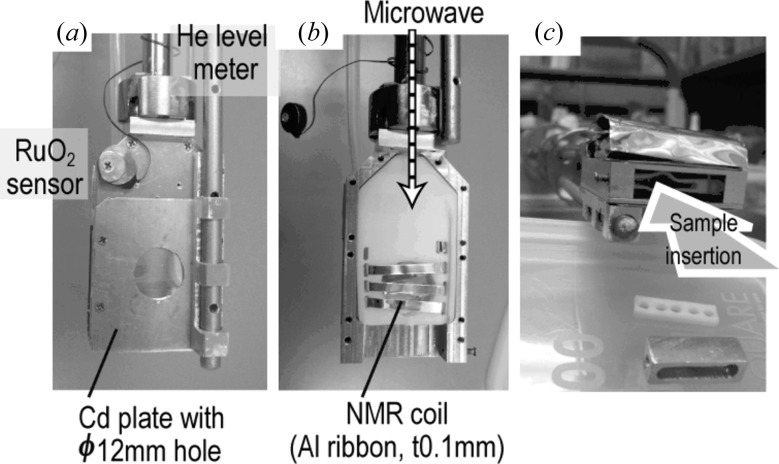
The sample cell of the DNP cryostat. Further detail for parts (*a*) to (*c*) is given in the text.

Fig. 4 ▸(*a*) shows a photograph of the sample cell. On the upstream side, a Cd plate with a 12 mm diameter hole is fixed. A thin (0.1 mm) aluminium sheet is fixed inside to prevent microwave leakage. The aluminium case has several holes for circulating liquid He; the diameter of these holes is 0.5 mm, which is much less than the 94 GHz microwave wavelength (3 mm). Fig. 4 ▸(*b*) shows a photograph of the sample cell when the upstream side cover is removed. Inside the aluminium case, a polytetrafluoroethylene (PTFE) part supports a three-turn NMR coil made of 0.1 mm-thick aluminium plate for *P*_H_ evaluation. *P*_H_ is evaluated using the continuous-wave NMR circuit, which is described later in §3.1. As shown in Fig. 4 ▸(*c*), a sheet sample (14 × 14 × 1 mm) is inserted from the bottom into the NMR coil. After insertion of the sample, the aluminium block supporting the sample bottom is fixed by screws. Microwave radiation (94 GHz) is generated by the Gunn oscillator fixed over the top plate of the cryostat, which irradiates the sample through a stainless steel pipe of length 1 m and inside diameter 6 mm, filled with a PTFE rod. The specifications of the DNP cryostat are summarized in Table 3 ▸.

### 2.4. SANS   

**Figure 5:**
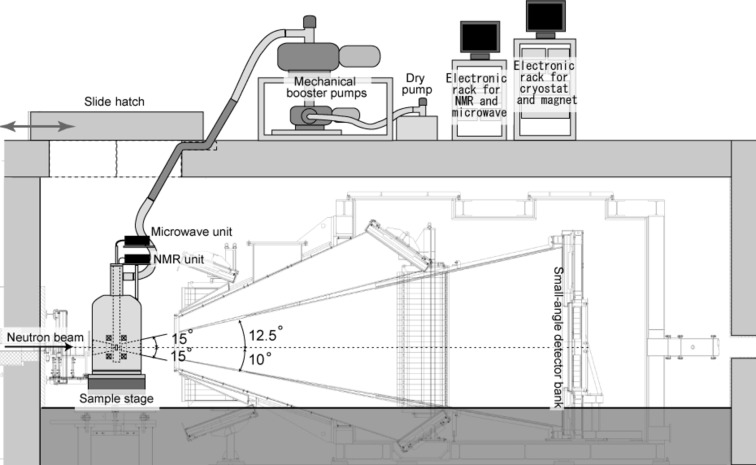
The DNP experimental layout on BL15 TAIKAN of the MLF at J-PARC.

TOF-SANS experiments were performed on the TAIKAN instrument (BL15) (Shinohara, Suzuki *et al.*, 2009 ▸; Shinohara, Takata *et al.*, 2009 ▸; Takata *et al.*, 2015 ▸) at the MLF of J-PARC. The TAIKAN instrument is equipped with a magnetic supermirror polarizer, composed of an Fe/Si multilayer (4*Q*_c_). Fig. 5 ▸ shows a schematic diagram of the device allocations on the SANS instrument. The roof of the shielding room has a sliding hatch covering the sample stage. Through this sliding hatch, the DNP cryostat was introduced onto the sample stage. The DNP cryostat was originally designed for SANS-J-II at JRR-3 and cannot fully cover the detectors of TAIKAN, having detectors for neutrons with wider scattering angles (2θ > 15°). The roof of the shielding room has two trenches under the sliding hatch. Through these trenches, pipes and cables were pulled out. The pipes are necessary to evaporate liquid ^4^He and to supply ^4^He gas. The cables are for energizing the superconducting magnet, monitoring the signals from the liquid helium level meter and the pressure and temperature sensors, measuring the NMR signals, and supplying voltage to the microwave generator. The mechanical booster pump unit and the two electronic racks were placed on the roof of the shield room. Consequently, we remotely controlled the magnetic field, sample temperature, NMR measurements and microwave irradiation.

## 3. Experimental results and discussion   

### 3.1. Proton NMR   

**Figure 6:**
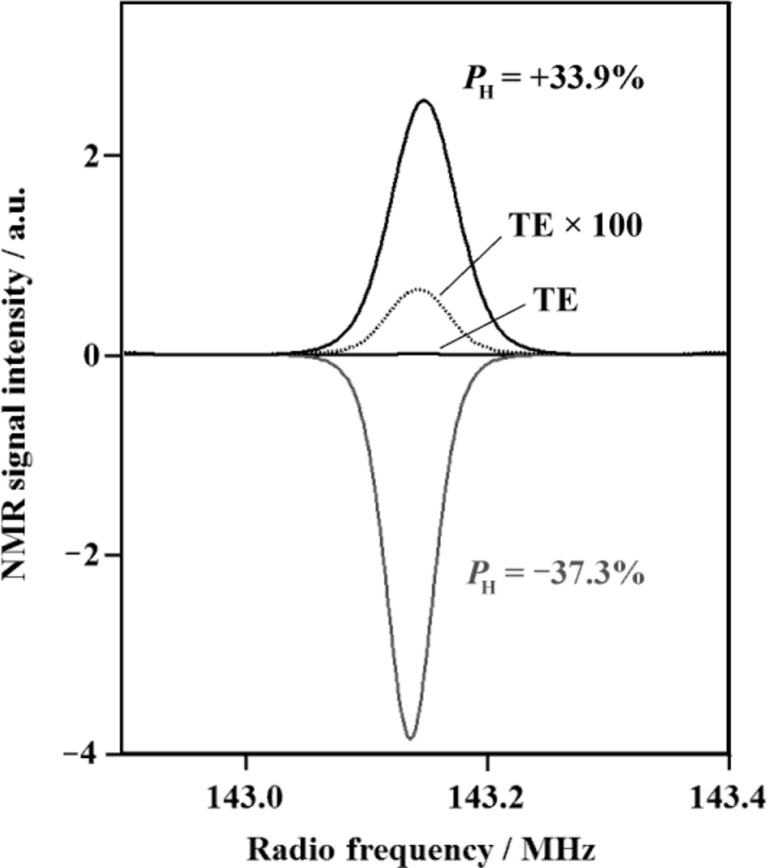
Proton NMR signal enhancement by DNP. TE denotes the signal observed at a thermal equilibrium of 3.35 T and 4.2 K (*P*_H_ = 0.082%). The sample was a 25 m*M* TEMPO-doped SBR rubber without filler.

Prior to SANS experiments with proton spin polarization, it is important to investigate the proton spin polarization behaviour of the TEMPO-doped rubber specimens using proton NMR. Fig. 6 ▸ shows the results of proton NMR. The integrated NMR signal was calibrated in order to coincide with the thermal equilibrium *P*_H_ = 0.082% at 3.35 T and 4.2 K, which is given according to equation (3). Then by NMR, we can evaluate the bulk *P*_H_ averaged over a whole specimen.

**Figure 7:**
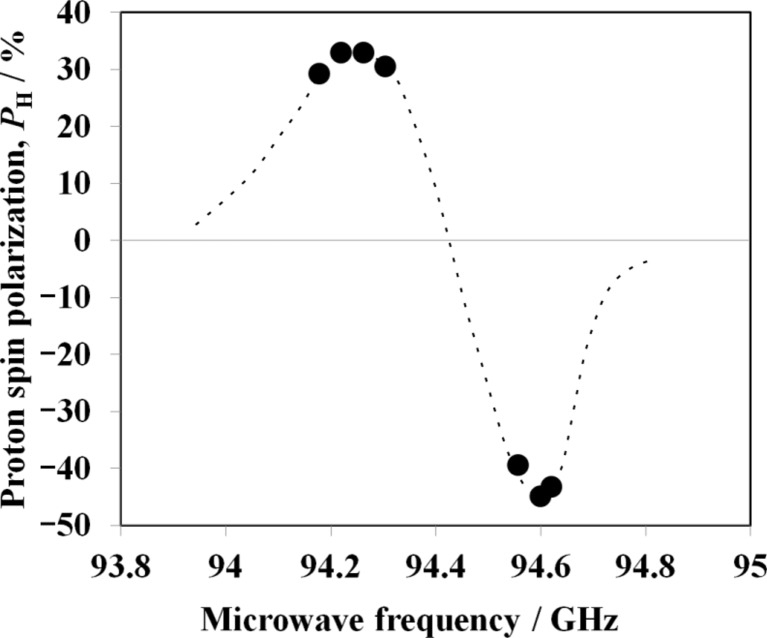
The microwave frequency dependence of proton spin polarization. The sample was a 25 m*M* TEMPO-doped SBR rubber without filler.

Fig. 7 ▸ shows the bulk *P*_H_ as a function of microwave frequency. By tuning the microwave frequency, the bulk *P*_H_ is converted from positive to negative. The microwave frequency can easily be controlled by the voltage supplied to the Gunn oscillator. The bulk *P*_H_ also depends not only on the microwave frequency but also on the sample temperature.

**Figure 8:**
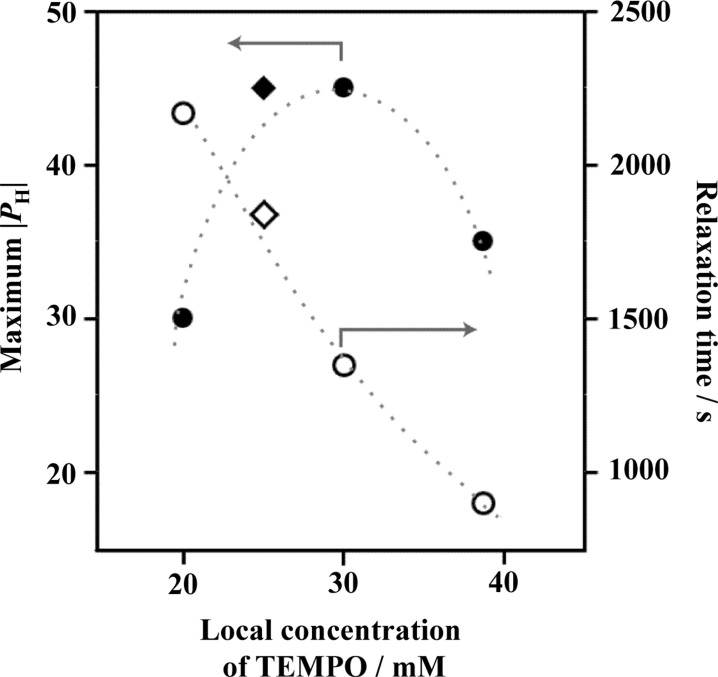
The maximum achievable |*P*_H_| for silica-filled SBR rubber samples as a function of local concentration of TEMPO.

Fig. 8 ▸ shows the bulk *P*_H_ determined as a function of TEMPO concentration. At 30 m*M*, |*P*_H_| reaches a maximum value. Fig. 8 ▸ also shows the proton spin relaxation time. As the TEMPO concentration increases, the spin relaxation time decreases monotonically owing to the increase in magnetic field fluctuation which is caused by TEMPO radicals.

**Figure 9:**
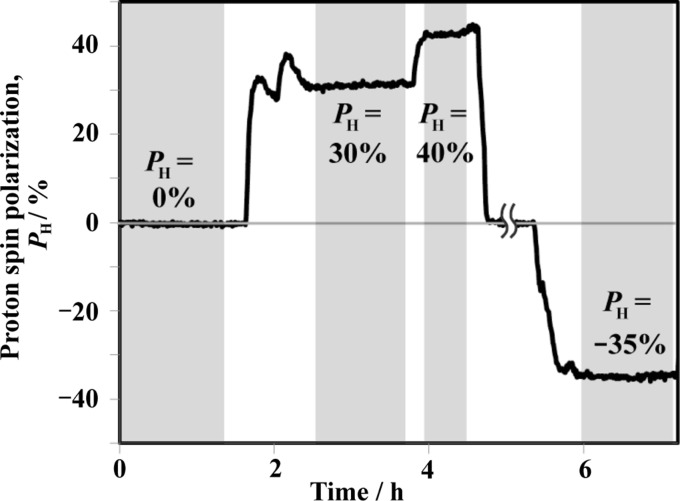
The time evolution of *P*_H_ evaluated by NMR during the SANS experiment on SBR/SP. The SANS measurements were performed during the periods shown in grey.

Fig. 9 ▸ shows the time profile of the bulk *P*_H_ during the SANS experiment on the binary mixture (SBR/SP). Each SANS measurement was performed when the bulk *P*_H_ was kept constant, as indicated by the shaded areas in Fig. 9 ▸. From 1.8 to 2.2 h, the bulk *P*_H_ was swept in order to search for the contrast matching condition by changing the microwave frequency. The bulk *P*_H_ quickly responded to the microwave frequency tuning (the time constant was 150–200 s). The fast response of *P*_H_ is convenient for SANS experiments.

In the time region with no shading, the bulk *P*_H_ changed continuously. Therefore, the coherent scattering length, and in turn the scattering contrast, also varied continuously. For such dynamic behaviours, the event–data format, which is commonly used in TOF-SANS experiments, is advantageous for extracting data from favourable time domains after the experiment.

### 3.2. SANS for the binary mixture (SBR/SP)   

#### 3.2.1. SANS without polarization   

**Figure 10:**
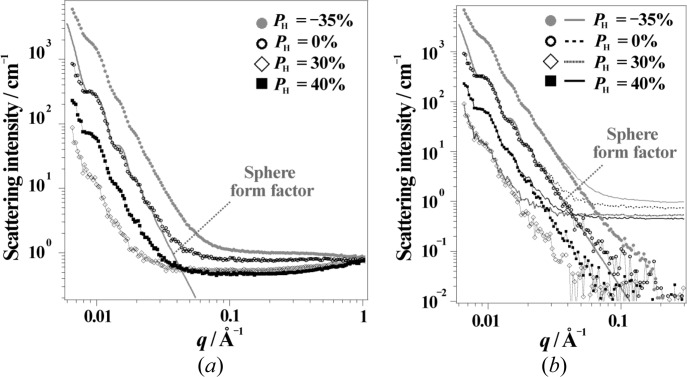
The SANS intensity of SBR/SP. In the profiles in panel (*a*), neutrons in the full wavelength range (1 < λ < 7.6 Å) were used. In the profiles in panel (*b*), neutrons in a limited wavelength range (4 < λ < 7.6 Å) were used. In panel (*b*), the symbols represent the profiles after subtracting the incoherent scattering. The thick grey profiles in panels (*a*) and (*b*) were calculated using the spherical form factor with radius 610 ± 40 Å.

Fig. 10 ▸(*a*) shows SANS obtained for the binary mixture (SBR/SP) without polarizing proton spins (*P*_H_ = 0%). The SANS intensity is shown as a function of the magnitude of the scattering vector *q* [*q* = (4π/λ)sin(θ/2), where λ and θ are the wavelength of the neutrons and the scattering angle, respectively]. In the low-*q* region, scattering maxima originating from the form factor of the SPs were observed. The scattering curves (thick grey curves in Figs. 10 ▸ *a* and 10 ▸ *b*) were reproduced using equations (5)–(7) [Disp-formula fd7] and considering a smearing effect for the BL15 collimation geometry:
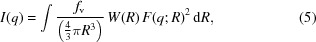
where
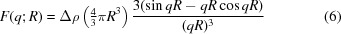
and
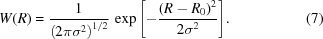
In equation (5), *f*_v_ is the volume fraction of spherical particles. In equation (6), *F*(*q*; *R*) is the scattering amplitude for a spherical particle of radius *R*, and Δρ is the scattering length density difference between the spherical particles and the surrounding medium. *W*(*R*) in equation (7) is a Gaussian function for the radius distribution, where *R*_0_ is the average radius and σ is the radius dispersion. As a result of our evaluations, we obtained *R*_0_ = 610 Å and σ = 40 Å. At that time, we calibrated SANS on the absolute intensity scale (cm^−1^) using the known volume fraction of SP.

#### 3.2.2. SANS under polarization   

**Figure 11:**
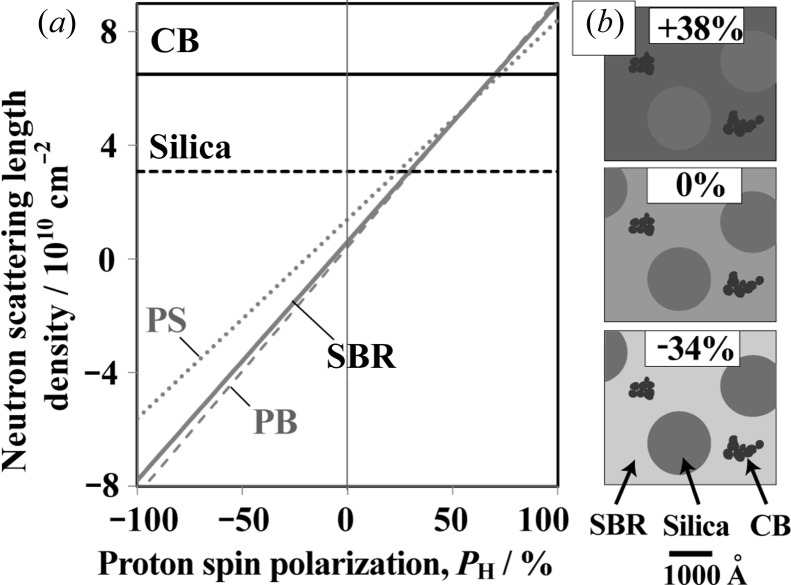
(*a*) Neutron scattering length density as a function of *P*_H_. (*b*) Schematics of the microstructure of SBR/SP/CP. The degree of shading reflects the neutron scattering length density.

As seen in Fig. 10 ▸, the intensity of SANS at low *q* increased at negative polarization *P*_H_ = −35%. For positive polarization, the scattering intensity decreased at *P*_H_ = 30% and increased again at *P*_H_ = 40%. In order to examine the scattering intensity depending on the bulk *P*_H_, we evaluated the coherent scattering length density of SBR according to equation (1) and the chemical composition of SBR. Simultaneously, we separately evaluated the coherent scattering length density for polystyrene (PS) and polybutadiene (PB) monomers, which compose the SBR chain. Note that the SANS scattering intensity is proportional to the square of the difference in the coherent scattering length density. We found that the matching point between silica and SBR appears at *P*_H_ = 30% (Fig. 11 ▸ *a*).

As shown in Fig. 10 ▸(*a*), at high *q* between 0.1 and 0.3 Å^−1^, the SANS exhibited *q*-independent behaviour. It can readily be seen that the scattering intensity in this *q* region has decreased with increasing *P*_H_. This is consistent with the description for incoherent scattering according to equation (2). However, the influence of the imperfect neutron polarization on the incoherent scattering should be noted. If we postulate *P*_N_ = 0 in equation (2), we obtain 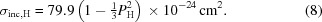
In Fig. 1 ▸(*b*), σ_inc,H_ with *P*_N_ = 0 is shown by the dashed line, indicating clearly different behaviour from the case with *P*_N_ = 1.

**Figure 12:**
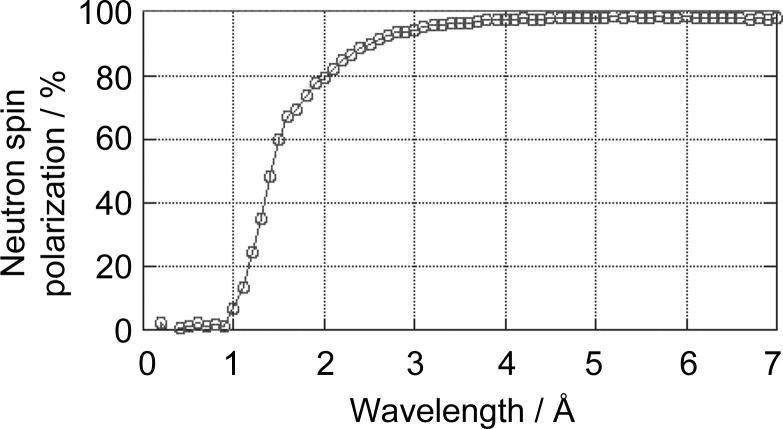
Neutron spin polarization as a function of neutron wavelength, λ, obtained for the BL15 supermirror polarizer.

In the TOF-SANS experiments, we utilized neutrons with a wide λ range simultaneously. The shorter-λ neutrons contribute to the higher-*q* region, whereas the longer-λ neutrons contribute to the lower-*q* region. Fig. 12 ▸ shows the *P*_N_ provided at TAIKAN as a function of λ. *P*_N_ has decreased with decreasing λ for λ < 4 Å, and become almost zero for λ < 1 Å.

Polarized neutrons are used effectively in polarization analysis for separating coherent and incoherent scattering. In this technique, by examining the spin-flip and non-spin-flip contributions, we can separate the coherent and incoherent scattering contributions. In the separation process, the λ dependence of *P*_N_ can easily be corrected.

However, in the case of spin contrast variation, the λ dependence of *P*_N_ affects not only the incoherent scattering length but also the coherent scattering length. Therefore, in the following analysis in this study, we only use data with 4 < λ < 7.6 Å, where *P*_N_ > 97% is satisfied. The results are shown in Fig. 10 ▸(*b*). The observed *q* region is limited (*q* < 0.3 Å^−1^) because we excluded the shorter-*λ* contribution.

#### 3.2.3. Transmission under polarization   

**Figure 13:**
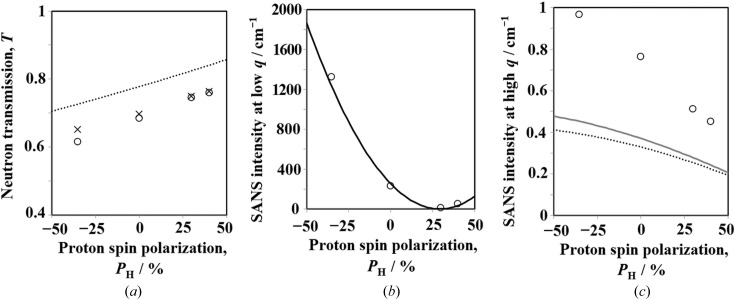
(*a*) Neutron transmission, (*b*) scattering intensity at low *q* (0.01 Å^−1^) and (*c*) scattering intensity at high *q* (0.3 Å^−1^), all as a function of *P*_H_ for SBR/SP. In panel (*a*), the circles and crosses denote the results for neutrons with λ = 6 Å and λ = 4 Å, respectively. The dotted line is the calculated exp(−Σ_tot_ *t*). In panel (*c*), the dotted and solid grey lines are the calculated Σ_inc_/(4π) and [exp(Σ_inc_ *t*) − 1]/(4π*t*), respectively.

The transmission (*T*) increased with increasing *P*_H_, as shown in Fig. 13 ▸(*a*). *T* is given by 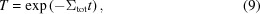
where *t* is the sample thickness and Σ_tot_ is given by
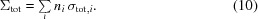
Here, *i* is the index for labelling the nuclear species, *n*_*i*_ is the number density for the labelled nuclear species and σ_tot,*i*_ is the total cross section (the sum of coherent scattering, incoherent scattering and absorption cross sections) of the labelled nuclear species. For protons, the total scattering cross section (σ_tot,H_) is given by
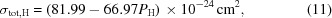
and for SBR/SP, Σ_tot_ = 4.49 − 3.48*P*_H_ cm^−1^. In Fig. 13 ▸(*a*), *T* calculated by equation (9) is indicated by the dotted line. The transmission determined experimentally is consistent with the evaluation by equation (9), although the values are slightly high. The difference might be attributed to sample thickness distribution or multiple and inelastic scattering.

#### 3.2.4. In the low-*q* region   

The scattering intensity at low *q* (*q* = 0.01 Å^−1^) is shown as a function of *P*_H_ in Fig. 13 ▸(*b*). In the low-*q* region, the coherent scattering originating from silica particles is dominant. The solid line in Fig. 13 ▸(*b*) exhibits a contrast factor in between those of the silica and SBR phases [(ρ_SP_ − ρ_SBR_)^2^], where ρ_SP_ is the scattering length density of silica (3.08 × 10^10^ cm^−2^) and ρ_SBR_ is the scattering length density of SBR [(0.62 + 8.39*P*_H_) × 10^10^ cm^−2^]. The evaluated solid line agrees well with the experimental results.

#### 3.2.5. In the high-*q* scattering region   

The scattering intensity at high *q* (*q* = 0.3 Å^−1^) is shown as a function of *P*_H_ in Fig. 13 ▸(*c*). In this high-*q* region, incoherent scattering (*I*_inc_) is dominant because the coherent scattering intensity from silica particles decreases drastically with increasing *q*. *I*_inc_ is given by
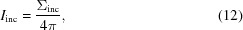
where Σ_inc_ is the sum of the incoherent scattering cross section per unit volume,
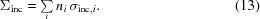
Here, *i* is the index for labelling nuclear species, *n*_*i*_ is the number density for the labelled nuclear species and σ_inc,*i*_ is the incoherent scattering cross section of the labelled nuclear species. Using σ_inc,H_ in equation (2) for SBR/SP, equation (13) transforms to 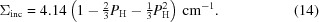
The calculated results are shown in Fig. 13 ▸ *c* by the dotted line, which does not reproduce the experiments well. By considering multiple scattering effects, *I*_inc_ is given by the following equation (Shibayama *et al.*, 2005 ▸):
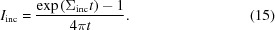
In Fig. 13 ▸(*c*), the solid grey line is calculated using equation (15) and a thickness *t* = 0.056 cm. It is closer to the experimental result, but still lower. This discrepancy might be attributed to the coherent scattering contribution that still exists in the high-*q* region. As shown in Fig. 11 ▸, the coherent scattering length densities of the PS and PB composing SBR deviate from each other for negative *P*_H_. Thus, local concentration fluctuations between PS and PB might give rise to coherent scattering, even at high *q*.

### 3.3. SANS for the ternary mixture (SBR/SP/CP)   

**Figure 14:**
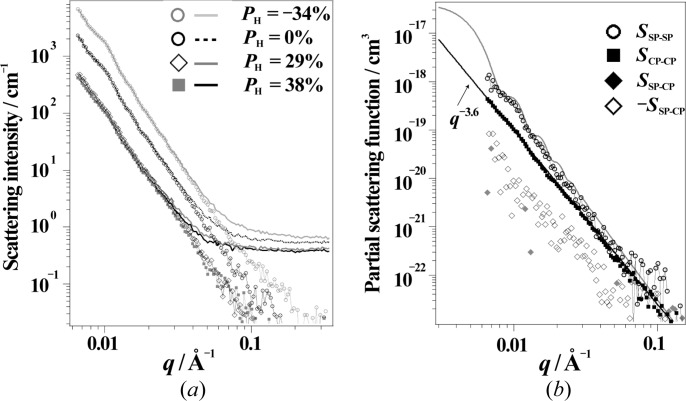
(*a*) The SANS profiles for SBR/SP/CP. The scattering profiles after subtraction of the incoherent scattering are denoted by symbols. (*b*) The separated partial scattering functions (*S*_SP–SP_, *S*_CP–CP_ and *S*_SP–CP_). The grey curve is the calculated spherical form factor with the parameters determined for SBR/SP.

Fig. 14 ▸(*a*) shows the SANS results obtained for the ternary mixture system (SBR/SP/CP). According to the discussion in §3.2 for the data reduction, we employed the limited wavelength range of 4 < λ < 7.6 Å, which gives *P*_N_ > 97%. As expected for a ternary mixture, the *q* dependence of the SANS varies significantly with changing proton spin polarization; the scattering maxima due to silica particles were observed for *P*_H_ = 0% and *P*_H_ = −34%, whereas they disappeared for *P*_H_ = 29% and *P*_H_ = 38%. As already shown in Fig. 11 ▸, the scattering length densities of silica and SBR match at *P*_H_ = 30%. Around the matching point, the CB contribution was observed more clearly.

#### 3.3.1. Partial scattering function decomposition   

The SANS intensity for the ternary mixture is given by the sum of three partial scattering functions [*S*_SP–SP_(*q*), *S*_CP–CP_(*q*) and *S*_SP–CP_ (*q*)] as follows:
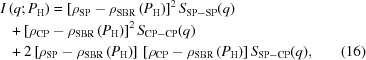
where the partial scattering functions are weighted by a contrast factor. In this equation, ρ_SP_, ρ_CP_ and ρ_SBR_ correspond to the neutron scattering length densities of the silica, CB and SBR phases, respectively: ρ_SP_ = 3.08 × 10^10^ cm^−2^, ρ_CP_ = 6.50 × 10^10^ cm^−2^ and ρ_SBR_ = (0.62 + 8.39*P*_H_) × 10^10^ cm^−2^. Only ρ_SBR_ depends on *P*_H_, because SBR contains hydrogen. *S*_SP–SP_(*q*), *S*_CP–CP_(*q*) and *S*_SP–CP_(*q*) are defined by the following equation:
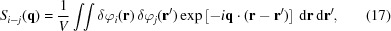
where the subscript *i* or *j* labels one of the components (SP, CP or SBR) and δφ_*i*_(**r**) indicates the fluctuation in the volume fraction of component *i* at position **r**. We observed the SANS at four different *P*_H_ (Fig. 14 ▸ *a*). Then, the *I*(*q*; *P*_H,*i*_) for different *P*_H,*i*_ (*i* = 1 to 4) are described by 
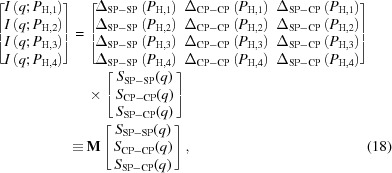
where the 4 × 3 matrix in the first line is denoted by **M**. The matrix elements are composed of contrast factors, as follows:
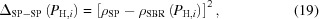


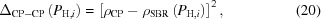


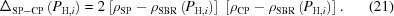
Depending on our experiments, **M** forms a non-square matrix (the number of experiments corresponds to that of the rows). Therefore, instead of a simple inverse matrix, we need to employ the method of the Moore–Penrose pseudo-inverse matrix, **M** ^+^, defined as follows:
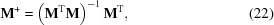
where **M** ^T^ is the transposed matrix of **M**. **M** ^+^ is known to give the shortest-length least-squares solution for equation (18), as follows:
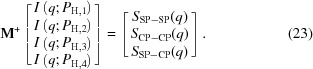
We evaluated **M** ^+^ for the four different *P*_H_ (0, 29, 38 and −34%) and the obtained plots of *S*_SP–SP_, *S*_CP–CP_ and *S*_SP–CP_ are shown by the symbols in Fig. 14 ▸(*b*). The partial scattering function of silica, *S*_SP–SP_, agreed well with the spherical form factor, the same as that for SBR/SP. The partial scattering function of CB, *S*_CP–CP_, indicated a power-law function of *q* ^−3.6^, deviating from the Porod law (*q* ^−4^) (Porod, 1951 ▸). This originates from surface structure of CB. The result agrees with reports for CB-filled rubber specimens (Koga *et al.*, 2005 ▸, 2008 ▸). The decomposition into partial scattering functions was successfully achieved. The cross-correlation term between silica and CB, *S*_SP–CP_, was also determined. *S*_SP–CP_ is negligibly small compared with *S*_SP–SP_ and *S*_CP–CP_.

## 4. Concluding remarks   

In this paper, we have reported the first attempt to use DNP and contrast variation SANS experiments on model mixtures for industrial tyres conducted at the MLF of J-PARC. We performed TOF-SANS experiments, employing neutrons with a wide λ range, which causes imperfect neutron polarization and variations in the coherent and incoherent scattering lengths. By carefully eliminating the effect of imperfect neutron polarization, separation of the partial scattering functions was successfully demonstrated for the ternary system SBR/SP/CP.

## Figures and Tables

**Table 1:** Proton and electron spin polarization at thermal equilibrium (3.35 T)

Temperature	Proton spin polarization	Electron spin polarization
300 K	0.001%	0.7%
4.2 K	0.082%	48%
1.2 K	0.30%	95%

**Table 2:** Specimen composition in vol.% DCP is dicumyl peroxide, TBBS is *N*-*tert*-butyl-2-benzothiazyl sulfenamide, DPG is 1,3-diphenylguanidine and Acc. denotes accelerator.

Specimen	S-SBR	Silica	CB	Stearic acid	Silane-coupling agent	DCP	Acc. TBBS	Acc. DPG
SBR/SP	84.6	10.0	0.00	1.87	1.49	0.71	0.62	0.66
SBR/SP/CP	82.7	10.0	2.00	1.83	1.50	0.69	0.60	0.65

**Table 3:** Specification of the DNP cryostat

Magnetic field	3.35 T, parallel with neutron beam, inhomogeneity < 0.5 × 10^−4^ (relative)
Specimen temperature	1.2 K (single-shot), ^4^He evaporation cryostat
Microwave	94 GHz Gunn oscillator
NMR circuit	144 MHz, continuous-wave, frequency-sweep
